# Single-cell sequencing reveals the origin and the order of mutation acquisition in T-cell acute lymphoblastic leukemia

**DOI:** 10.1038/s41375-018-0127-8

**Published:** 2018-04-18

**Authors:** Jolien De Bie, Sofie Demeyer, Llucia Alberti-Servera, Ellen Geerdens, Heidi Segers, Michaël Broux, Kim De Keersmaecker, Lucienne Michaux, Peter Vandenberghe, Thierry Voet, Nancy Boeckx, Anne Uyttebroeck, Jan Cools

**Affiliations:** 1 Center for Human Genetics, KU Leuven, Leuven, Belgium; 2 Center for Cancer Biology, VIB, Leuven, Belgium; 3 Leuvens Kanker Instituut (LKI), KU Leuven – UZ Leuven, Leuven, Belgium; 4 Department of Pediatric Hemato-Oncology, UZ Leuven, Leuven, Belgium; 5 Department of Hematology, UZ Leuven, Leuven, Belgium; 6 Wellcome Trust Sanger Institute, Hinxton Cambridge, UK; 7 Department of Oncology, KU Leuven, Leuven, Belgium; 8 Department of Laboratory Medicine, UZ Leuven, Leuven, Belgium

## Abstract

Next-generation sequencing has provided a detailed overview of the various genomic lesions implicated in the pathogenesis of T-cell acute lymphoblastic leukemia (T-ALL). Typically, 10–20 protein-altering lesions are found in T-ALL cells at diagnosis. However, it is currently unclear in which order these mutations are acquired and in which progenitor cells this is initiated. To address these questions, we used targeted single-cell sequencing of total bone marrow cells and CD34^+^CD38^−^ multipotent progenitor cells for four T-ALL cases. Hierarchical clustering detected a dominant leukemia cluster at diagnosis, accompanied by a few smaller clusters harboring only a fraction of the mutations. We developed a graph-based algorithm to determine the order of mutation acquisition. Two of the four patients had an early event in a known oncogene (*MED12*, *STAT5B*) among various pre-leukemic events. Intermediate events included loss of 9p21 (*CDKN2A/B*) and acquisition of fusion genes, while *NOTCH1* mutations were typically late events. Analysis of CD34^+^CD38^−^ cells and myeloid progenitors revealed that in half of the cases somatic mutations were detectable in multipotent progenitor cells. We demonstrate that targeted single-cell sequencing can elucidate the order of mutation acquisition in T-ALL and that T-ALL development can start in a multipotent progenitor cell.

## Introduction

T-cell acute lymphoblastic leukemia (T-ALL) is a common childhood malignancy caused by clonal proliferation of immature T cells. Analysis of T-ALL genomes with various technologies has revealed that 10–20 protein-altering mutations are typically present at diagnosis [1–3]. *CDKN2A/2B* and *NOTCH1* are the most frequently affected genes in T-ALL, with 60% of T-ALL patients showing activation of the NOTCH1 signaling pathway and up to 80% harboring deletions and/or mutations inactivating the *CDKN2A/B* genes at chromosome 9p [4, 5]. The majority of T-ALL cases is also characterized by chromosomal rearrangements resulting in the ectopic expression of the transcription factors TAL1, TLX1, TLX3, NKX2-1 or HOXA [4]. Other pathways that are frequently mutated in T-ALL include the JAK/STAT (Janus kinase/signal transducer and activator of transcription) and RAS (Rat Sarcoma oncogene) signaling pathways [1, 3, 6, 7]. Several *JAK1*, *JAK2, JAK3*, *NRAS* and *KRAS* mutations have been described, as well as mutations in *IL7R* and *DNM2*, which also result in activation of the JAK/STAT pathway [1, 8, 9]. Fusion genes may lead to hyperactivation of kinases, as is the case with the *NUP214*–*ABL1* fusion or various *JAK2* and other tyrosine kinase fusions [10, 11]. Next-generation sequencing studies have further identified mutations in ribosomal proteins *RPL5*, *RPL10* and *RPL22*, as well as in various transcriptional and epigenetic regulators, such as *PHF6*, *CNOT3, PRC2* and many others [2, 7, 12]. Deep sequencing revealed that many of these mutations are present at subclonal levels and that leukemia is therefore heterogeneous at presentation [1, 13–16].

Despite this detailed information on the various mutations that are implicated in T-ALL and their clonal frequency, next-generation sequencing cannot discriminate between mutations co-occurring in the same cell or in different cells at low frequency. In addition, it remains unknown in which cells driver mutations first present and whether they occur in a specific or random order. To obtain such information accurately, a single-cell approach is indispensable. Over the past years, single-cell sequencing technologies have tremendously improved, enabling us to obtain information on mutations, expression and chromatin structure. Cells can be isolated manually, with laser capture microdissection or by flow cytometric sorting and automated microfluidic devices [17–19]. A critical step for single-cell DNA and RNA analysis remains the amplification step, because a single cell only contains a limited amount of DNA and RNA transcripts. Many different DNA amplification techniques exist, each with specific advantages and disadvantages [17, 20, 21]. For RNA amplification, tag-based or full-length amplification methods are available. Tag-based methods are biased towards the 3’ or 5’ end of the transcripts and therefore primarily suited for gene expression profiling [17, 22, 23].

Over the last few years, several research groups have used single-cell DNA sequencing to evaluate the clonal structure of normal and diseased tissue samples, but only limited data are available for hematological malignancies and T-ALL has not yet been covered [24–27]. In this study, we used single-cell DNA and RNA sequencing to determine the clonal heterogeneity of primary T-ALL samples, and exploited these data to determine the order in which mutations are acquired. Moreover, by applying single-cell sequencing to sorted progenitor cells, we also identified the genomic lesions initiating T-ALL in multipotent progenitors.

## Methods

Diagnostic and remission bone marrow (BM) samples were collected from children diagnosed with T-ALL at Leuven’s University Hospital on protocol S57176 approved by the Ethical Committee University Leuven. Written informed consent was obtained from every patient in accordance with the Declaration of Helsinki. Viably frozen cells were thawed at 37 °C followed by suspension in phosphate-buffered saline (PBS) supplemented with 10% fetal calf serum. Cells were washed and prepared for single-cell isolation on a small C1 DNA sequencing chip (IFC, 5–10 µm, Fluidigm). Alternatively, cells were filtered (40 µm) and sorted as single cells in 96-well plates, containing 4 µL PBS per well, using Aria III or Aria IIu (BD). Single-cell RNA sequencing was performed on the Chromium system (10× Genomics). Full methods are available as supplementary data.

## Results

### Identification of somatic variants using bulk whole genome and transcriptome sequencing

Whole-genome sequencing (WGS) and RNA sequencing was performed on BM samples obtained at diagnosis and remission from four childhood T-ALL cases (Supplemental table S1). All patients had normal karyotypes and a high tumor burden in the diagnostic BM. Patient characteristics are described in Table 1. For each patient, an average of 10 coding variants were identified in the bulk tumor sample. In addition, we detected several fusion genes, large deletions and somatic mutations present in 5’- and 3’-UTR regions and splice sites (Supplemental Fig. 1). All patients had at least one mutation in *NOTCH1*, whereas three out of four also had a deletion or mutation leading to inactivation of *CDKN2A* and/or *CDKN2B*. Two patients (XB41 and XB47) showed rearrangements at the *NKX2-1* locus. These rearrangements were complex and involved T-cell receptor genes as fusion partners. Patient XB37 carried both a *STIL-TAL1* fusion and a *LMO2* juxtaposition to the *TRD* locus. Interestingly, we also detected a novel *TCF7-SPI1* fusion gene in patient X09, who also carried a *NRAS* mutation. Although this article was in preparation, similar SPI1 fusion genes were described in pediatric T-ALL cases[28].

**Table 1:** Patient characteristics and somatic genomic lesions in 4 T-ALL cases

	X09	XB37	XB41	XB47
Gender	Male	Male	Female	Male
Age (y)	6	12	9	9
WBC (×10^9^/L)^a^	691	195	32	35
% Bone marrow blasts^b^	93	87	89	79
Immunophenotype	Medullar	Immature	Cortical	Cortical
Karyotype	46,XY[1]	46,XY[12]	46,XX[7]	46,XY[14]
FISH	9p21(*CDKN2A*) loss 14q11 rearrangement	*STIL-TAL1* *TRD-LMO2*	14q11 rearrangement	9p21 (*CDKN2A*) loss
Mutations or indels detected by WGS and RNA sequencing^c^	NOTCH1 F1606ins MED12 P22L NRAS G12D TBL1XR1 D85E SDK1 V1300M KLF9 P31L	NOTCH1 V1605ins CDKN2A D68Stop STAT5B N642H BCL11B A732ins SLCO3A1 654Fs FAT2 3522Fs	NOTCH1 L1600P RPL10 R98S RPL26L1 R115Q CMTM5 R8W NOTUM S406L ACOX1 S482N PCDHA10 E342K	NOTCH1 L1600P NOTCH1 Y2490Stop RPL10 R98S PHIP P259L CNOT3 R745ins N4BP2 N1670S SLC6A18 T91M PPP4C D54K
Chromosomal rearrangements detected by WGS and RNA sequencing^c^	del(9)(p21p21) *TCF7-SPI1*	*LMO2* *STIL-TAL1* fusion	*NKX2-1* del(14)(q11q11)	*de*l(9)(p21p21) *NKX2-1*

^a^ White blood cell count in the peripheral blood at diagnosis

^b^ Blast counts were determined by microscopy of bone marrow smears and confirmed with flow cytometry

^c^ Somatic mutations/indels/rearrangements in known oncogenes/tumor-suppressor genes identified by combined whole-genome sequencing and RNA sequencing data analysis of the bulk diagnostic and remission samples

**Fig. 1:**
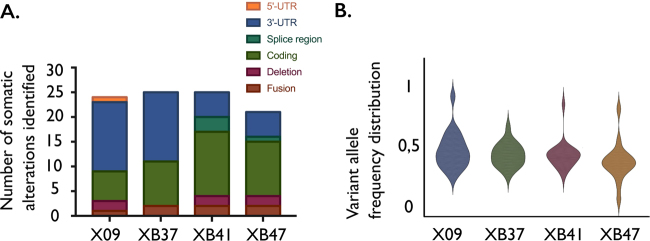
Somatic variant identification by bulk sequencing of four primary T-ALL samples. (A) Overview of the number and different types of somatic variants identified in each T-ALL patient by bulk whole-genome and RNA sequencing, subsequently used for targeted single-cell sequencing. (B) Violin plots illustrating the variant allele frequency distributions of the bulk mutations identified per patient

We selected on average 24 tumor alterations per patient (coding variants, non-coding variants and chromosomal rearrangements) (Figs. 1a, b, Supplemental tables S2-S5). From all single-nucleotide polymorphisms (SNPs) detected, we selected 32 heterozygous SNPs that were shared by the four cases (confirmed as heterozygous by Sanger sequencing). These 32 SNPs were used for quality control assessment of the single-cell analysis (Supplemental table S6). We next developed specific primer sets to enable targeted amplicon sequencing of the selected SNPs and somatic alterations for each patient, as previously described [27, 29].

### Single-cell targeted sequencing and quality control

**Fig. 2:**
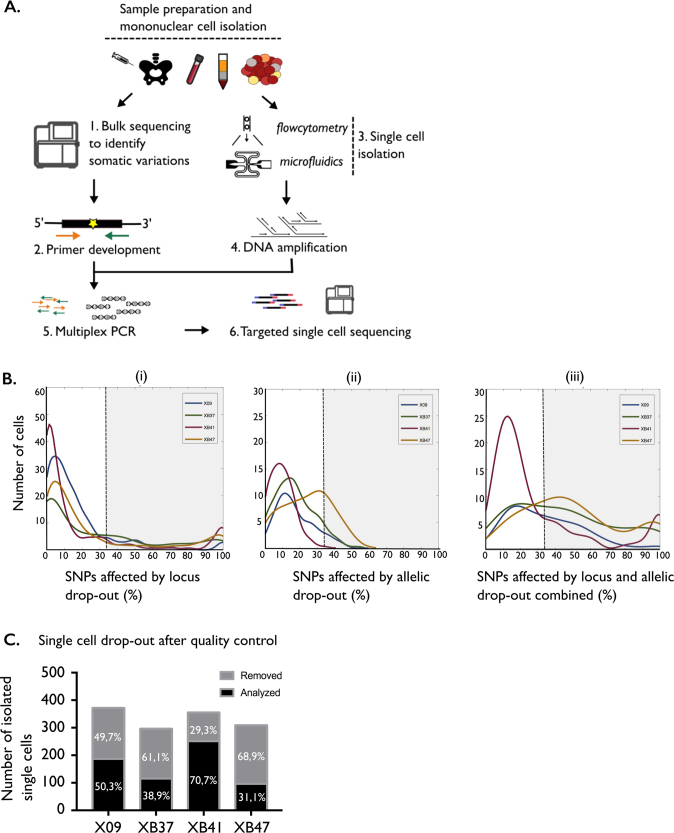
Targeted single-cell sequencing of four primary T-ALL samples. (A) Schematic workflow of the protocol describing the targeted single-cell sequencing. (B) Histograms of the locus drop-out rates (i), allelic drop-out rates (ii) or locus and allelic drop-out rates combined (iii). Quality control consisted of removing all cells with more than one-third of SNPs affected by locus and allelic drop-out combined, indicated by the gray area in panel (iii). (C) Bar chart of the absolute numbers of single leukemic cells isolated per patient together with the percentage of cells retained for analysis after quality control

We isolated on average 333 single leukemic cells per patient from the mononuclear cells obtained from a BM sample with > 75% blast cells. After amplification of the single-cell genomes, we applied the patient-specific primer sets and sequenced the regions of interest (Fig. 2a, Supplemental Fig. 2). We compared the bulk variant allele frequencies (VAFs) with the combined single-cell VAFs for each mutation and found overall good correlation, except for patient XB47 where the single-cell VAFs were consistently lower than bulk VAFs for all mutations. Detailed investigation of the single-cell data revealed high number of normal blood cells, which may have been absent in the (distinct) sample used for bulk DNA analysis (Supplemental Fig. 3, Supplemental table S7).

To exclude low-quality cells, we looked at the locus and allelic drop-out (ADO) rate for the heterozygous SNPs in each cell (Supplemental Fig. 4). We defined the locus drop-out (LDO) rate as the percentage of SNPs with <4 reads [27, 30]. High LDO rates indicate that many SNPs in a cell have low coverage, indicating that larger regions of the genome were not amplified. ADO exemplifies that heterozygosity of a SNP might be lost if only one of the alleles is amplified. Following quality cut-offs of previous studies, only cells with less than one-third of SNPs affected by combined LDO and ADO were considered of sufficient quality and used for further analysis (Fig. 2b, Supplemental Methods)[24, 27]. Of the total 1332 isolated single leukemic cells, 649 fulfilled these criteria. There was a striking difference in the number of cells complying with our quality control measures among patients (Fig. 2c).

### Targeted single-cell sequencing reveals up to four T-ALL clusters at diagnosis

**Fig. 3:**
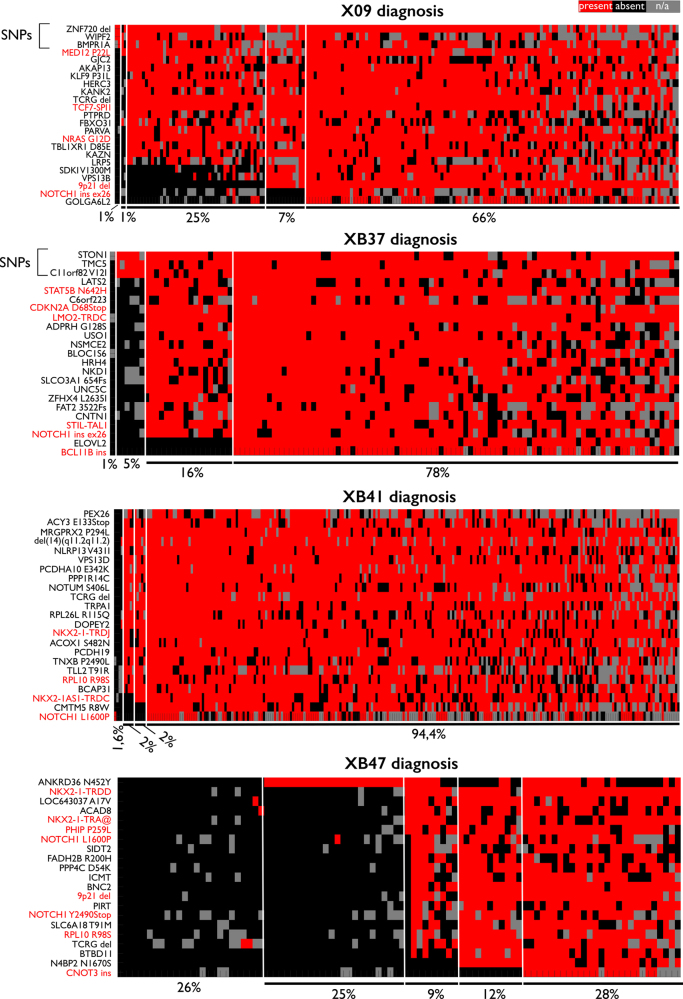
T-ALL patient samples have limited heterogeneity at presentation. Heatmaps of the somatic variations detected per patient: X09 (*n* = 187 cells), XB37 (*n* = 115 cells), XB41 (*n* = 251 cells) and XB47 (*n* = 96 cells). Columns represent single cells, rows represent the somatic variations. The order of both the cells and the variations is based on hierarchical clustering with the Jaccard distance as metric. Presence of a variation is indicated in red, absence in black, whereas gray represents variations with <10 reads (i.e., no data available). Gene names from known oncogenic drivers are colored red. Percentages indicate the relative number of cells attributed to each clone

Jaccard hierarchical clustering was applied to the targeted single-cell data, resulting in the identification of two to four clusters per sample (Supplemental table S8, Supplemental Fig. 5). Every patient harbored a highly mutated dominant cell cluster, comprising 28–94% of all single cells, accompanied by a number of smaller clusters carrying fewer mutations (Fig. 3).

Patient X09 had a highly mutated dominant cluster (66% of all cells), accompanied by a very small cell cluster (1%) harboring a previously described pathological *MED12* mutation (COSM1124623, http://cancer.sanger.ac.uk/cosmic/mutation/overview?id=1124623). Two intermediate clusters comprising 25% and 7% of the cells were also detected. Both clusters had acquired, among others, a *TCF7–SPI1* fusion and *NRAS* G12D mutation, whereas cells from the smallest cluster had gained an extra deletion of *9p21*. 1% of the cells lacked mutations and likely represent normal BM cells.

In patient XB37, a major cluster was detected comprising 78% of the single cells. This cluster was highly mutated and accompanied by two smaller cell clusters of 5% and 16%, carrying respectively only a few SNPs or all mutations except the *ELOVL2* mutation and *BCL11B* insertion. We also detected one wild-type cell in this sample.

Patient XB41 had the most homogeneous leukemia, harboring one major cluster with all mutations and two small clusters, each representing only 2% of the cells, that had acquired almost all events, except for the *NKX2–1AS1–TRDC* fusion and/or *CMTM5* and *NOTCH1* mutations. A wild-type population comprising 1.6% of the cells was also detected.

Finally, the major cluster in patient XB47 contained 28% of all cells and carried all the mutations, whereas three smaller clusters of 25%, 9% and 12% of the cells harbored only an *ANKRD36* mutation or all mutations except the *N4BP2* mutation and/or *CNOT3* insertion, respectively. Another 26% of the cells did not have any of the investigated alterations and likely represent normal cells, correlating with the blast count of 79% in this patient.

Overall, these data are compatible with a stepwise hierarchy because each cell cluster harbored more mutations than the last. In all four T-ALL patients, we could clearly detect some of these ‘ancestor’ clusters at diagnosis, indicating that these must have a sufficiently high proliferation capacity and are not completely outcompeted by the dominant leukemia cluster.

### Single-cell RNA sequencing reveals transcriptional uniformity of T-ALL cells

**Fig. 4:**
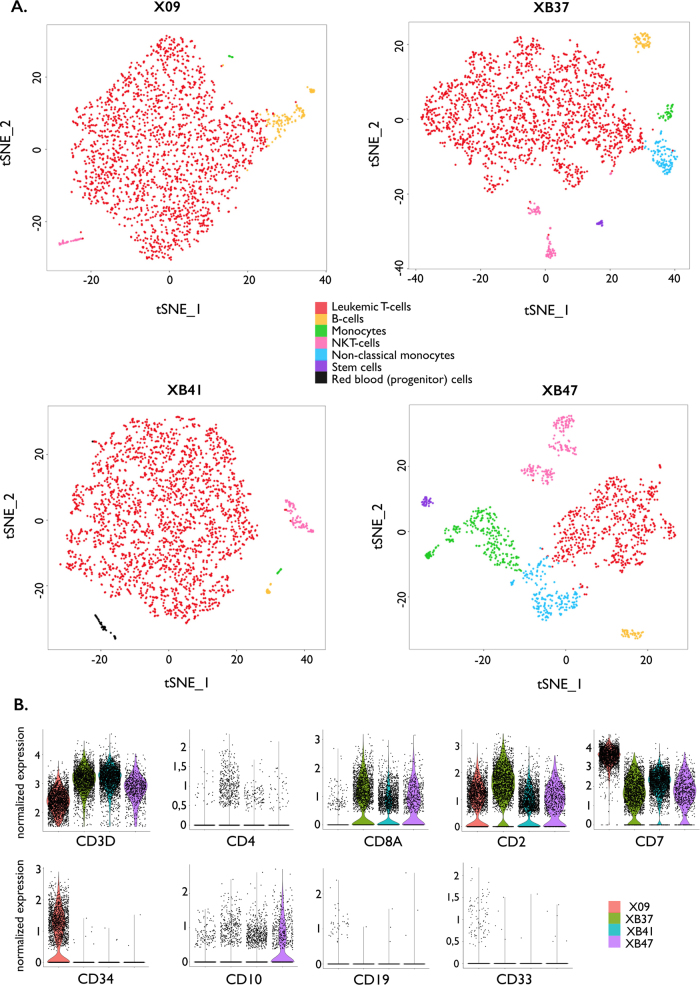
Single-cell RNA sequencing reveals transcriptional uniformity of T-ALL cells. **a** tSNE analysis and cluster allocation for the single cells per patient. Cluster allocation is described in more detail in Supplemental Methods and Supplemental Fig. 6.** b** Violin plots showing the normalized expression of several cluster of differentiation markers for the leukemic T cells in each patient. Expression levels correspond with the immunophenotype established with flow cytometry at the time of diagnosis (data not shown). CD19 and CD33 expression represent negative controls

To further investigate the heterogeneity of the T-ALL samples, we applied single-cell RNA sequencing and searched for differences in gene expression levels within the T-ALL cells. An average of 2074 cells per patient were analyzed (Supplemental methods, Supplemental Fig. 6, Supplemental table S7). Leukemic cells consistently clustered together, indicating limited transcriptional heterogeneity among the T-ALL cells. Other clusters represented normal B cells, (non-classical) monocytes, natural killer T cells, stem cells and even few hemoglobin-producing red blood (progenitor) cells (Fig. 4a). Clustering of the CD3-positive T cells alone disclosed three clusters for case XB47, whereas the three other cases did not show different clusters (Supplemental Fig. 7). Analysis of all single cells or all CD3-positive cells from the four patients together, revealed that leukemic cells clustered per patient, whereas normal cells clustered together (Supplemental Fig. 8−9).

The gene expression of several surface markers was evaluated in the leukemic cells (Fig. 4b) and matched with the immunophenotype at diagnosis (data not shown). Similar to the targeted DNA sequencing data, patient XB47 contained only 47% of leukemic T cells in the investigated sample (Supplemental table S7).

### T-ALL mutations can initiate in CD34^+^CD38^−^ multipotent progenitor cells

To determine if mutations and chromosomal aberrations in T-ALL are acquired in hematopoietic stem cells and early multipotent progenitors, we isolated 175 single CD34^+^CD38^−^ progenitor cells from the diagnostic and remission samples using flow cytometry (Fig. 2a, Supplemental Fig. 10A) [31].

**Fig. 5:**
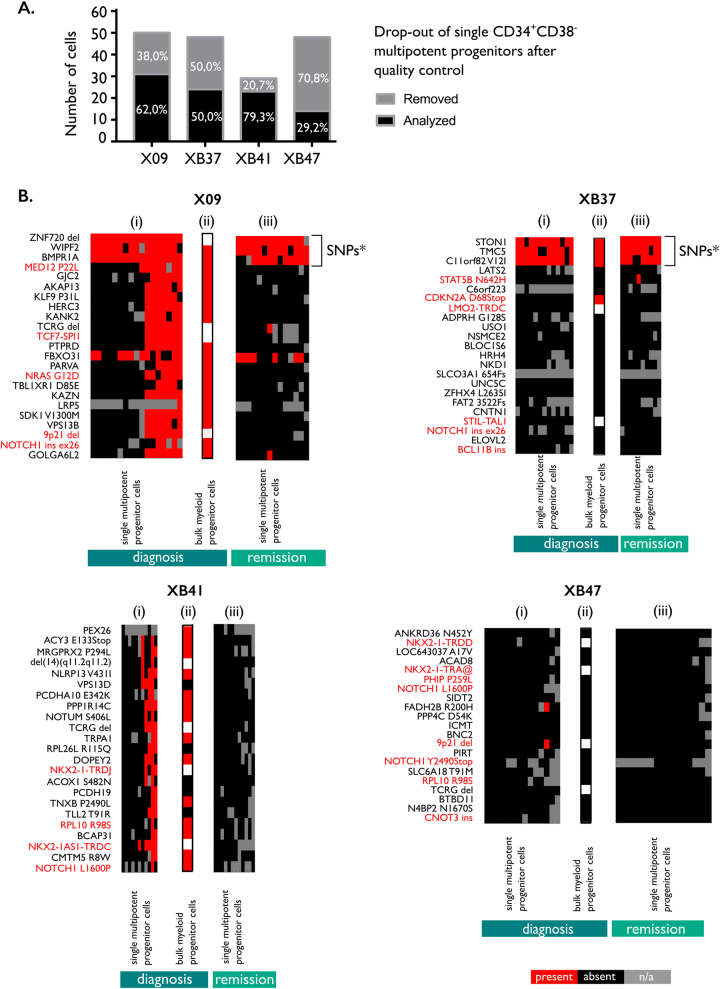
Multiple mutations can be present in multipotent progenitor cells. **a** Bar chart of the absolute numbers of single CD34^+^CD38^-^ multipotent progenitor cells isolated per patient and the percentage of cells accepted for analysis after quality control. **b** Heatmaps of the variations in single CD34^+^CD38^-^ multipotent progenitor cells isolated from patient X09, XB37, XB41 or XB47 taken at diagnosis (i) and at remission (iii). Sanger sequencing was performed on bulk DNA extracted from 2000 to 5000 myeloid progenitor cells sorted from the diagnostic samples (ii) to confirm the presence of the mutations found in the multipotent progenitor cells at diagnosis. Deletions and fusion genes were not evaluated in the bulk myeloid progenitor DNA to prevent false-positive results caused by few contaminating leukemic cells, and are therefore colored white in the graph. The order of both the cells and the variations is based on hierarchical clustering with the Jaccard distance as metric. *These variations were initially considered somatic mutations, based on the WGS results of the remission sample. However, we could confirm the presence of these SNPs with PCR on the bulk remission samples

The CD34^+^CD38^−^ progenitors underwent identical targeted sequencing and quality control procedures as described above (Fig. 5a). In addition, we sorted bulk committed myeloid progenitors (CD34^+^CD135^+^CD33^+^) from the diagnostic samples to determine the presence of mutations in this myeloid population (Supplemental Fig. 10B).

In two of the four T-ALL patients (X09, XB41), CD34^+^CD38^−^ progenitor cells were identified that showed a highly mutated profile. The majority of these mutations were also detected in diagnostic myeloid committed progenitor cells, which is compatible with the majority of mutations being acquired in a stem cell or multipotent progenitor cell in these patients. However, these highly mutated multipotent progenitors were eradicated in both patients after achieving remission. In contrast, for the other two T-ALL patients (XB37, XB47) very few mutations were detected in the CD34^+^CD38^−^ cells and myeloid progenitor cells, indicating that the majority of these mutations were acquired in progenitors already committed to the lymphoid lineage (Fig. 5b).

### Targeted single-cell sequencing can determine the order of mutation acquisition in T-ALL

To determine the order in which mutations were acquired during T-ALL development, we applied a newly developed graph-based algorithm to our single-cell data. This algorithm enumerates all possible orders of events and scores them according to the evidence found in the experimental single-cell data. Information from both leukemic cells and diagnostic CD34^+^CD38^−^ progenitor cells was taken into account, although removal of the multipotent progenitors from the algorithm had no impact on the resulting mutational hierarchy (data not shown).

**Fig. 6:**
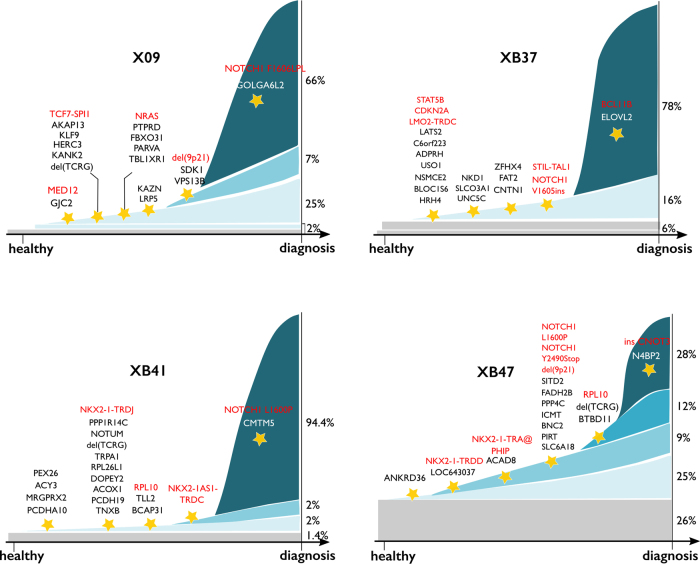
Single-cell data illuminate the mutational hierarchy in T-ALL patient samples. The order of mutation acquisition based on the newly developed graph-based algorithm for patient X09, XB37, XB41 and XB47. The algorithm evaluated all single-cell information available from both diagnostic leukemic and CD34^+^CD38^-^ multipotent progenitor cells and stipulated the most probable order of events. Its output (including the 100 most probable order of events per patient) is provided in Supplemental table S9. Percentages on the right represent clones detected at diagnosis per patient, whereas the stars represent different steps in mutation accumulation. Events that happened together or are closely related in time are represented by their respective gene names and written above each star

In all four patients, early mutations happened in genes of unknown significance, whereas patient X09 and XB37 also had an early event in a known oncogene, *MED12* and *STAT5B* respectively. Intermediate events included inactivation of *CDKN2A/B* and deletions in T-cell receptor genes due to T-cell receptor rearrangements, and also the parallel acquisition of fusion genes. Interestingly, mutations in *NOTCH1* were relatively late events in three of the four patients, happening after the bulk of mutations and fusion genes were acquired (Fig. 6, Supplemental table S9). The late acquisition of the *NOTCH1* mutations and the occurrence of chromosomal rearrangements could not have been deduced easily from the VAFs obtained by bulk sequencing (Supplemental table S10).

## Discussion

In this study, we performed targeted single-cell sequencing on 1507 single cells isolated from the BM of four childhood T-ALL patients. We detected up to four leukemia cell clusters at diagnosis with the dominant cluster comprising 28–94% of all cells. Our data is in line with observations for B-ALL and AML, for which a similar level of heterogeneity was described [25, 27]. We detected a stepwise hierarchy between the clusters in the T-ALL samples. This result corresponds with evidence found in multiple myeloma, where early ancestor clones were also detectable at diagnosis [26]. Moreover, single-cell RNA sequencing suggests that T-ALL cells are also highly similar at the gene expression level. The limited heterogeneity detected in our childhood T-ALL cases may have important implications for treatment, as tumor heterogeneity could influence the risk for relapse. Larger studies that include temporal/spatial data will be needed to obtain a more complete view on the heterogeneity of T-ALL at diagnosis and during treatment, and to determine if the degree of heterogeneity has prognostic value.

Importantly, by performing targeted sequencing on single CD34^+^CD38^−^ multipotent progenitor cells, we gained insight in the cell of origin of T-ALL. We compared sequence data from single CD34^+^CD38^−^ multipotent progenitor cells at diagnosis and at remission and compared these findings with mutations found in bulk myeloid progenitor cells isolated from the diagnostic samples. In two patients, we could detect most of the known oncogenic mutations in CD34^+^CD38^−^ multipotent progenitor cells and in myeloid progenitors, providing evidence that mutations in some T-ALL patients start to accumulate in multipotent progenitor/stem cells. After treatment, these events were no longer detectable in CD34^+^CD38^−^ progenitors, which is in line with long-term remissions for childhood T-ALL patients. These observations also recapitulate the importance of performing allogeneic stem cell transplantations for high-risk ALL patients. Autologous stem cell transplantation may indeed lead to relapse, in case the highly mutated multipotent progenitor cells are not eradicated before the procedure [32–34].

Our newly developed algorithm could infer the order in which mutations were acquired based on single-cell data. Early events included mostly genes of unknown significance, whereas fusion genes and loss of *CDKN2A/B* appeared later during leukemic development. Interestingly, mutations in *NOTCH1* were relatively late events in our patients. This confirms the finding of subclonal *NOTCH1* mutations in up to 43% of T-ALL patients in bulk sequencing studies [13, 35, 36]. Targeted sequencing with high read depth can be used to estimate mutational hierarchy from bulk sequencing data, but the lack of interpretable VAFs for chromosomal rearrangements in bulk sequencing data prevents accurate prediction on the order of those events. Single-cell sequencing overcomes this limitation because it provides information for every single-cell separately.

Patients can have distinct clinical presentations and treatment responses depending on the order at which mutations are acquired. This was recently demonstrated for patients with *JAK2* and *TET2* double mutated polycythemia vera, where the individuals who had first acquired a *JAK2* mutation had a higher risk of thrombosis and responded better to ruxolitinib than those who had first gained a *TET2* mutation [37]. *NOTCH1* is regarded as an interesting target for therapy in T-ALL [5], but our data indicate that it is typically a late mutation that is not necessarily present in all subclones, which may limit the therapeutic efficacy of targeting NOTCH1. Moreover, we show that, in some patients, mutations can start to accumulate in multipotent progenitor cells, illustrating the need for therapies that target these early hematopoietic cell states.

## Electronic supplementary material

supplementary methods

supplemental figure legends

Supplementary Figure 1

Supplementary Figure 2

Supplementary Figure 3

Supplementary Figure 4

Supplementary Figure 5

Supplementary Figure 6

Supplementary Figure 7

Supplementary Figure 8

Supplementary Figure 9

Supplementary Figure 10

Supplementary tables S1-S7

Supplementary table S8

Supplementary table S9

Supplementary table S10
