# Supplementary material for: Single-cell sequencing reveals the origin and the order of mutation acquisition in T-cell acute lymphoblastic leukemia
Source: Leukemia. 2018 Apr 18;32(6):1358–69. doi: 10.1038/s41375-018-0127-8 (PMC5990522; doi:10.1038/s41375-018-0127-8)
Supplement: Supplementary file 1 — supplementary methods [file 41375_2018_127_MOESM1_ESM.docx]

**Supplementary Methods**

*Patient Samples*

Selection of the four patients was based on the presence of both diagnostic and remission samples taken at 2 years after initial diagnosis. Mononuclear cells were isolated using Ficoll-Paque and viably frozen in 95% fetal calf serum and 5% DMSO.

*Whole Genome and Transcriptome Sequencing to identify Somatic Variants*

DNA and RNA were extracted from the diagnostic and remission BM samples (Maxwell, Promega) and prepared for sequencing using the KAPA Hyper Prep kit (Illumina). Libraries were sequenced on a HiSeq2500 with 125bp paired-end reads (Illumina). Poor quality reads were trimmed using fastq-MCF (ea-utils)^28^ and the quality was checked with fastQC (http://www.bioinformatics.babraham.ac.uk/projects/fastqc) before alignment with Bowtie2 (DNA)^29^ or Tophat2 (RNA)^30^ to human reference genome GRCh37. Duplicates were removed using Picard and we applied VarScan^31^ to identify true somatic mutations and indels between the leukemia and remission samples. We included somatic mutations with a VAF of at least 5% in the tumor sample, in order to allow detection of mutations in minor subclones. In addition, the relative difference between the VAF in the tumor and the VAF in the remission sample was required to be minimum 20% and significant based on Fisher’s exact test, and the VAF at remission was <5%. Furthermore, the mutations of interest were visually confirmed in IGV. Chromosomal translocations were determined in the DNA by BreakDancer^32^ and we used FusionCatcher^33^ to find gene fusions from the RNA data.

*Single-Cell Isolation using C1 Single-Cell Autoprep System*

Viably frozen cells from the selected patients were thawed in a 37°C incubator followed by suspension in phosphate buffered saline (PBS) supplemented with 10% fetal calf serum. Cells were washed and prepared for single cell isolation on a small C1 DNA sequencing chip (IFC, 5-10µm) according to Fluidigm’s instructions. After loading, each IFC was imaged to determine the single cell capture rate. The cells then underwent lysis and multiple displacement whole genome amplification (MDA) according to manufacturer’s instructions (Fluidigm). Two IFC’s were run for each patient, with exception of patient XB41.

*Single-Cell Isolation using Flow Cytometry*

Viably frozen cells from the selected patients were thawed in a 37°C incubator followed by suspension in PBS supplemented with 10% fetal calf serum. Cells were washed twice with PBS, counted with TC20 (Bio-Rad) and brought at a concentration of 2 million cells per mL. Cells were stained with Fixable Viability Stain 450 (BD Horizon) or propidium iodide to select for viable cells. For isolation of the single multipotent progenitor cells, CD34 PE-Cy7 (BD, 581) and CD38 FITC (eBioscience, HB7) antibodies were included in the staining mix. Cells were then filtered (40 µm) and sorted as single cells in 96-well plates, containing 4 µL PBS per well, using Aria III or Aria IIu (BD). Forward-side scatter were used to separate leukemic cells from normal, contaminating mononuclear cells in the BM sample. The single cells next underwent lysis and MDA whole genome amplification using Repli-G Single Cell kit (Qiagen). Plates could also be frozen immediately after sorting at -80°C for later analysis.

We isolated in total 1332 single cells for patient X09 (n=372), XB37 (n=296), XB41 (n=355) and XB47 (n=309).

*Investigation of Multipotent Progenitor and Myeloid Progenitor Cell Populations*

We used flow cytometry to isolate single CD34^+^CD38^-^ multipotent progenitor cells from the diagnostic and remission BM samples of each T-ALL patient (X09, n=50; XB37, n=48; XB41, n=29; XB47, n=48) and subjected them to the same targeted single cell sequencing analysis described above. In addition, we isolated 2000-5000 myeloid progenitor cells (CD34^+^CD135^+^CD33^+^) in bulk from the diagnostic samples using flow cytometry (ARIAIIu, BD and S3 Cell Sorter, Bio-Rad). Extracted and amplified DNA was obtained with REPLI-g Mini Kit (Qiagen), followed by standard PCR reactions using patient-specific primer sets and Sanger sequencing.

The following flow cytometry antibodies were used: anti-human CD34 (BD, 560710), anti-human CD38 (eBioscience, 11-0388-42), anti-human CD135 (Miltenyi Biotec, 130-111-665), anti-human CD33 (eBioscience, 11-0339-42).

*Targeted Single Cell Sequencing*

Primers were developed with Primer3plus and tested on bulk samples prior to use on the single cell amplified DNA. For large deletions and chromosomal translocations, we included primers targeting the abnormal and wild-type alleles. Single cell DNA samples underwent multiplex PCR on the Access Array System (Fluidigm) according to manufacturer’s guidelines, followed by standard PCR to add sample specific barcodes. Amplicons were run on a MiSeq using 160bp single-end reads (Illumina). FastQC (Babraham Bioinformatics, http://www.bioinformatics.babraham.ac.uk/projects/fastqc) and fastq-MCF^28^ were used for quality control and adaptor removal respectively, followed by read alignment to patient specific reference sequences, i.e. the references containing the wild-type and the mutated targeted regions only. Only regions with minimum 10 reads were taken into account and a mutation call was accepted when 5 or more reads, representing more than 2% of all reads, supported the variant.

*Determining locus and allelic drop-out*

Based on the bulk whole genome sequencing data, we selected randomly 40 potential heterozygous single nucleotide polymorphisms (SNPs) throughout the genome that were present in each T-ALL patient both at diagnosis and remission, of which 32 were confirmed to be heterozygous by Sanger sequencing. Primers targeting these heterozygous SNPs were added to the multiplex PCR assay. Single cells were considered heterozygous for these loci if the same base change could be identified in 0.5% to 99.5% of the reads. Locus drop-out occurred if less than 4 reads were detected for a certain location and allelic drop-out if no base change was identified (less than 0.5% of the reads) or if it was present in more than 99.5% of the reads. Cells were discarded from further analysis if locus and allelic drop-out combined exceeded 33.3% of all investigated SNPs.

*Hierarchical Clustering*

Both cells and mutations were hierarchically clustered based on the Jaccard distance on the discretized variant allele frequency values. Heatmaps were drawn from which the different clusters could be identified. Initially unassigned cells were appointed to the closest cluster. This analysis was performed with Matlab R2016b.

*Determining the Order of Mutation Acquisition*

To determine the order in which the somatic events, i.e. point mutations, indels and fusions, occurred, a graph-based algorithm was developed that enumerates all possible orders of events and scores them according to the evidence found in the experimental data. Cell states are modeled as nodes with a binary code representing which events have occurred or not. These nodes are then connected by links representing adding one or multiple events. Next, costs representing evidence found in the experimental data were added to the nodes. A possible order of events can now be represented as a path between the cell state with no events (binary code 00…0) and cell state with all events (binary code 11…1). Considering that the best path might not be the single optimal solution and that parallel events might occur, all possible paths were enumerated. This was realized by a depth-first recursive path finding algorithm. Subsequently, the paths with the lowest costs were combined, resulting in clustering of sequential events and identifying them as simultaneous. The output of this algorithm was compared to the initial clustering.

*Graph-based algorithm*

We constructed a graph in which the nodes represent cell states and the links represent adding one or multiple events. Consider a sample with N events, then each node can be described as a binary number with N positions x_1_… x_N_, where x_i_=1 represents that event I has happened yet, while x_i_ = 0 means that it has not yet occurred. As a consequence, the network will have 2^N^ nodes at most. Now, we can build a tree starting from node 00…0 and ending in node 11…1, in which each link represents one event occurring, i.e. a 0 in one node changing to a 1 in the next node. This means that we now have a graph with 2^N^ nodes and N.2^N-1^ links.

Next, we added node costs in this network, which represent the evidence found in the experimental data. For each cell, we transformed its state to a binary code and then added a unit cost to the corresponding network node. When a cell had NA-values for certain events, the cost was split over all nodes that might correspond to this cell state.

To minimize the search algorithm calculation time, a number of optimizations were applied. First of all, as the number of nodes in the network was exponential, while the number of cells was limited, we removed all nodes of the network for which we had no evidence in the network, i.e. the nodes which had a cost of zero. As this might lead to an unconnected network, we now needed to add links that represent the occurrence of multiple events at once. We therefore added all possible links, under the condition that a link can only be added if 0’s change to 1’s, but never the opposite. Second, we optimized the node costs. To deal with the fact that the experimental data is error prone, the Hamming distance between the cell data and the nodes had to be taken into account. If the experimental data was very similar to the node, a larger cost was added, while if the data was very dissimilar a smaller cost was added. For each data point (cell state) d to each node n the following cost was added: 1/(HD(d,n)+1), where HD(d,n) represents the Hamming distance between the data d and the node n.

To find all possible orders of events, we now wanted to enumerate all possible paths between node 00…0 and node 11…1. As we were convinced that the best path might not be the single optimal solution and to deal with the fact that there might be events happening in parallel, we wanted to enumerate all possible paths. For this, a depth-first recursive path finding algorithm was applied. Subsequently, the paths were ordered according to their cost, and the top paths could then be combined, which often came down to combining sequential events and identifying them as happening at once. The output of this algorithm, could then be compared to the initial clustering. This algorithm was validated with the data found in the publication of Gawad et al.^27^ and a similar clonal hierarchy could be identified as described in the paper. A JAVA implementation of this algorithm is freely available as an open source project on https://sourceforge.net/projects/labjaco-sceo/.

*Single cell RNA sequencing*

Viably frozen cells from the selected patients were thawed in a 37°C incubator followed by suspension in PBS supplemented with 10% fetal calf serum. Cells were washed five times with PBS, counted with TC20 (Bio-Rad) and brought at a concentration of 1 million cells per mL. Samples were analyzed on the Chromium system (10x Genomics) according to manufacturer’s instructions for an expected capture rate of 3000 single cells per patient.

*10x Genomics single-cell RNA-sequencing data analysis*

The Chromium-prepared sequencing data was demultiplexed and converted to fastq-files with the Cell Ranger software provided by 10x Genomics. The same software package was used for alignment, filtering and UMI counting. This raw count data was then analyzed with the R-package Seurat. Cells with too many genes or too many mitochondrial RNA were filtered out as these might represent doublets. The data was normalized using a global-scaling normalization method. Subsequently the most variable genes were identified and a linear dimensional reduction (i.e. a principal component analysis (PCA)) was performed on the variable genes. The principal components were then used in a graph-based clustering algorithm. We opted for a resolution parameter of 0,1. For visualization purposes a non-linear dimension reduction (tSNE) was performed and the tSNE-plots were colored according to the clusters determined in the previous step.

As we noticed that a lot of genes marking the difference between the clusters were cell cycle genes, we opted to regress the cell cycle effects out. To achieve this, we used the 6 cell cycle gene sets defined by Whitfield et al. (Mol.Biol.Cell, 2002), and regressed the average expression values out of these gene sets. We repeated the principal component analysis after the cell cycle regression step.

To identify different cell types in the analysis, the following cell markers were used:

| T-ALL-cells | CD3D, CD4, CD8A, IL2RA (CD25) |
| --- | --- |
| B-cells | CD19, CD79A, CD79B, MS4A1 (CD20) |
| NKT-cells | CD3D, NKG7, KLRB1 (CD161), NCAM1 (CD56), FCGR3A (CD16) |
| Monocytes | S100A8, S100A9, ITGAM (CD11B), CD14, FCGR3A (CD16) |
| Non-classical monocytes | CD14, FCGR3A (CD16), S100A8, S100A9, CX3CR1 |
| Red blood cells | HBA1, HBA2, HBB |
| Stem cells | CD34 |

*Data Access*

Sequence data has been deposited at the European Genome-phenome Archive (EGA), which is hosted by the EBI and the CRG, under accession number EGAS00001002830.
