# Supplementary material for: Single-cell sequencing reveals the origin and the order of mutation acquisition in T-cell acute lymphoblastic leukemia
Source: Leukemia. 2018 Apr 18;32(6):1358–69. doi: 10.1038/s41375-018-0127-8 (PMC5990522; doi:10.1038/s41375-018-0127-8)
Supplement: Supplementary file 2 — supplemental figure legends [file 41375_2018_127_MOESM2_ESM.docx]

**Supplemental Figure Legends**

**Suppl. Figure 1. Copy number alterations in the T-ALL patient samples**

Circos plot showing full genome coverage for the 4 primary T-ALL patients. Remission is indicated in grey, diagnostic samples in red.

**Suppl. Figure 2. Single-cell sequencing depth for heterozygous SNPs and somatic variants**

Histograms showing the number of reads per cell and position for the 4 primary T-ALL patients for the heterozygous SNPs used for quality control (A) and the somatic variants (B).

**Suppl. Figure 3. Variant allele frequencies comparison for bulk and combined single-cell data**

Scatterplots per patient illustrating the relation between the variant allele frequencies derived from the bulk WGS analysis and the calculated variant allele frequencies derived from the combined single cell data. Each dot represents a specific genetic lesion, the diagonal line represents perfect fit.

Patient XB47 shows consistently lower variant allele frequencies derived from the single cells compared to the bulk analysis, likely due to a difference in normal bone marrow cells in the sample used for bulk and for single cell analysis.

**Suppl. Figure 4. Targeted single-cell analysis for 32 heterozygous SNPs**

Heatmaps of the heterozygous SNPs selected for quality control per patient. Columns represent single cells, rows represent heterozygous SNPs. Homozygosity of a SNP is indicated in black, heterozygosity in red, while grey represents SNPs with no coverage.

**Suppl. Figure 5. Graphical representation of the Jaccard clustering algorithm**

Heatmaps of the Jaccard distances for the single cells from the 4 T-ALL patients showing intra-cluster homogeneity and between-cluster heterogeneity.

**Suppl. Figure 6. Clustering and cell type assignment based on single-cell RNA expression levels**

tSNE-plots for each patient showing initial clustering of the single cells before and after cell cycle regression and after cell type assignment. Each dot symbolizes a single cell.

Dotplots represent average expression of different cell type marker genes in the clusters before and after refinement by cell type assignment. Each dot represents a distinct marker gene. How darker a dot, the higher the average expression in the cluster for this marker, while larger dots indicate a higher percentage of cells in the cluster expressing this marker.

NKT = natural killer T-cells, NCM = non-classical monocytes (CD14^low^, CD16^high^), RBC = red blood (progenitor) cells

**Suppl. Figure 7. CD3 positive T-cells are very similar on the transcriptional level**

tSNE analysis and cluster allocation for CD3+ T-cells from the 4 T-ALL cases.

NKT = natural killer T-cells

**Suppl. Figure 8. Non-leukemic cells from distinct patients cluster together**

tSNE analysis and cluster allocation for all cells present in the 4 T-ALL patients. Clusters are colored according to the assigned cell type (A) or sample origin (B).

NKT = natural killer T-cells

**Suppl. Figure 9. Leukemic T-cells from distinct patients cluster separately**

tSNE analysis and cluster allocation for all CD3+ T-cells present in all 4 T-ALL patients.

NKT = natural killer T-cells

**Suppl. Figure 10. Isolation of multipotent progenitor and myeloid progenitor cells from the diagnostic leukemia samples**

Flow cytometric gating strategy to isolate CD34^+^CD38^-^ multipotent progenitor cells (A) and CD33^+^CD135^+^CD34^+^ myeloid progenitors (B) from the diagnostic samples of patient X09, XB37, XB41 and XB47.

SSC = side scatter
