# Supplementary figures and images for: Single-cell sequencing reveals the origin and the order of mutation acquisition in T-cell acute lymphoblastic leukemia
Source: Leukemia. 2018 Apr 18;32(6):1358–69. doi: 10.1038/s41375-018-0127-8 (PMC5990522; doi:10.1038/s41375-018-0127-8)

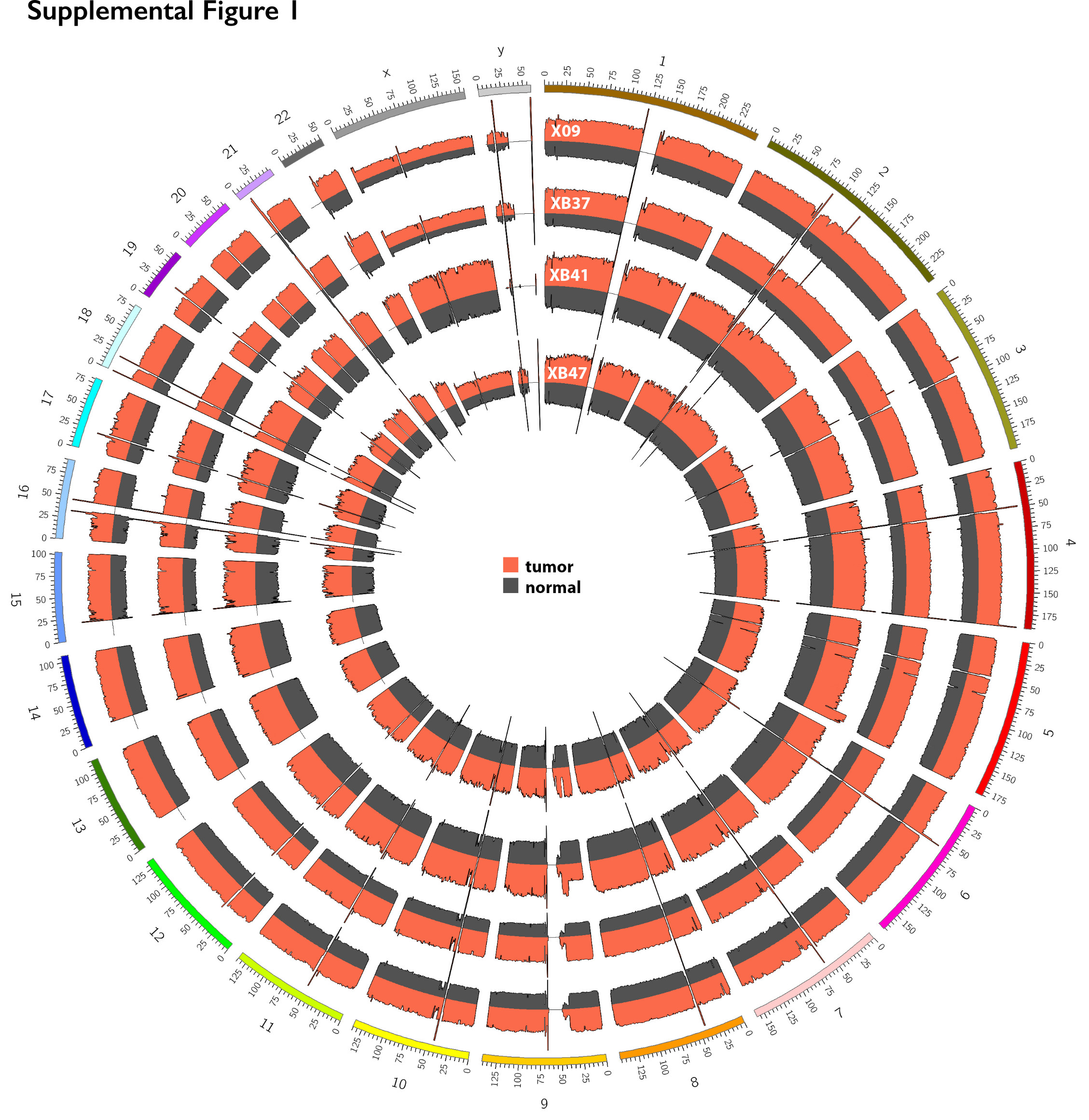

Supplement: Supplementary file 3 — Supplementary Figure 1 [file 41375_2018_127_MOESM3_ESM.jpg]

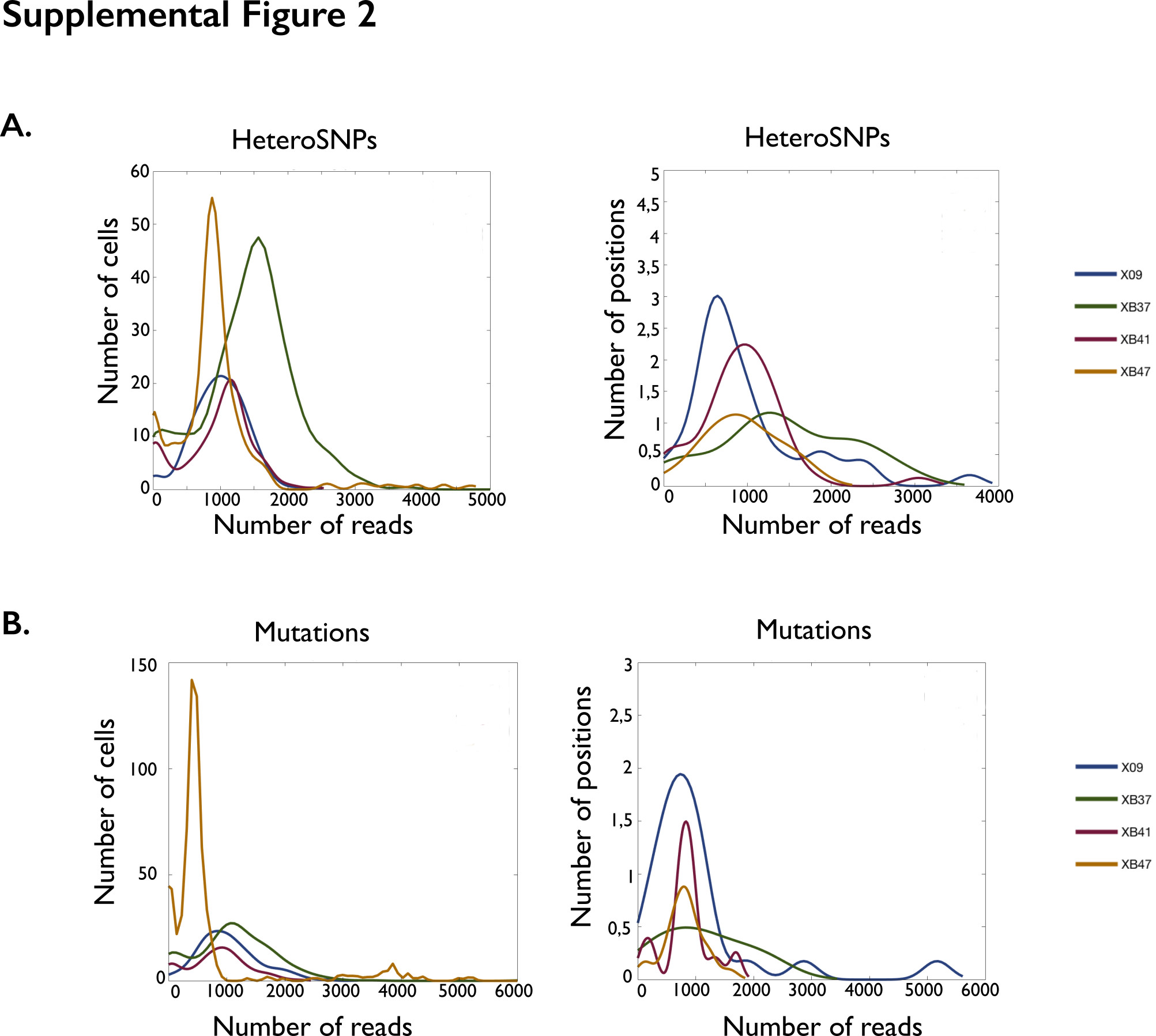

Supplement: Supplementary file 4 — Supplementary Figure 2 [file 41375_2018_127_MOESM4_ESM.jpg]

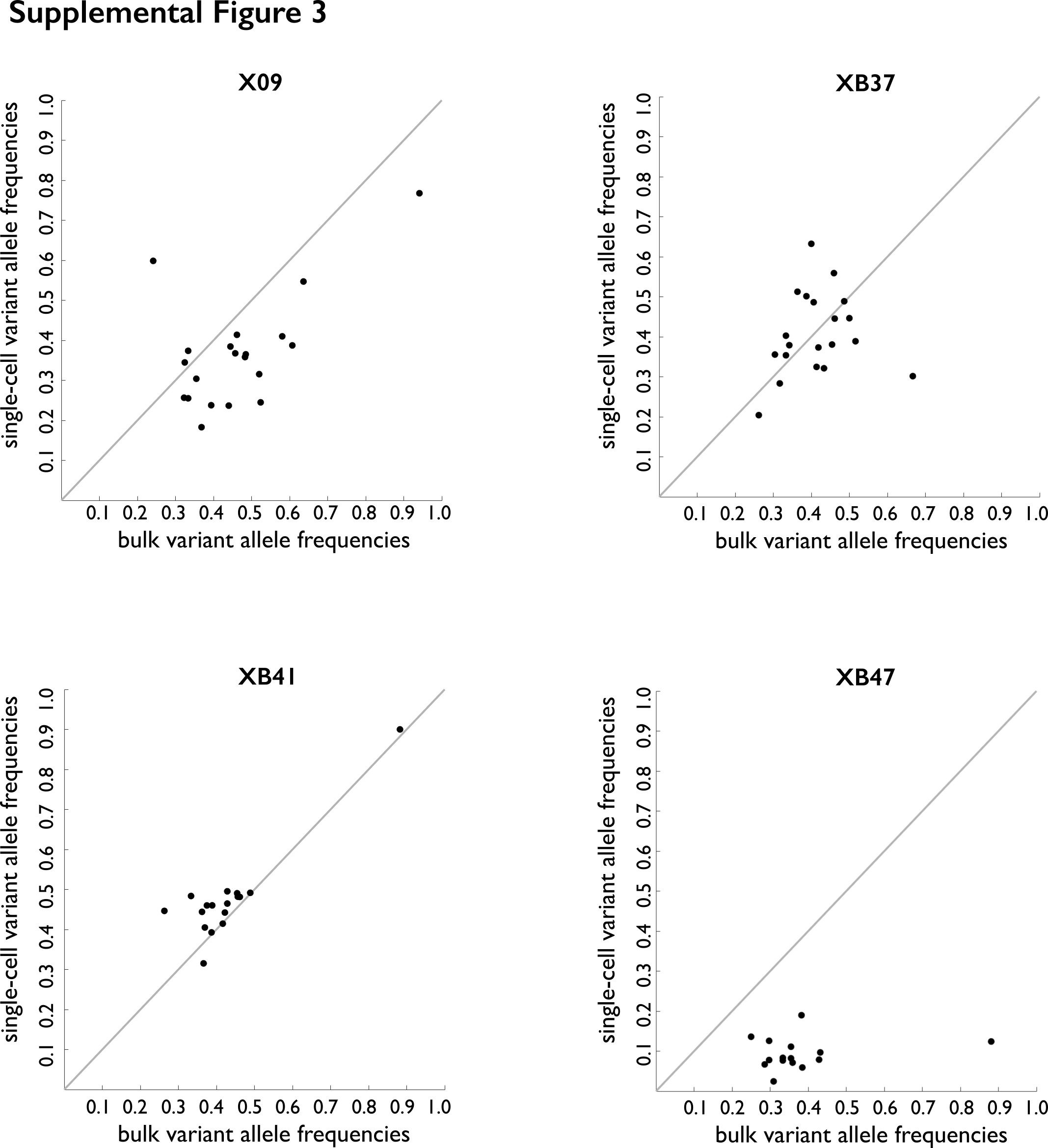

Supplement: Supplementary file 5 — Supplementary Figure 3 [file 41375_2018_127_MOESM5_ESM.jpg]

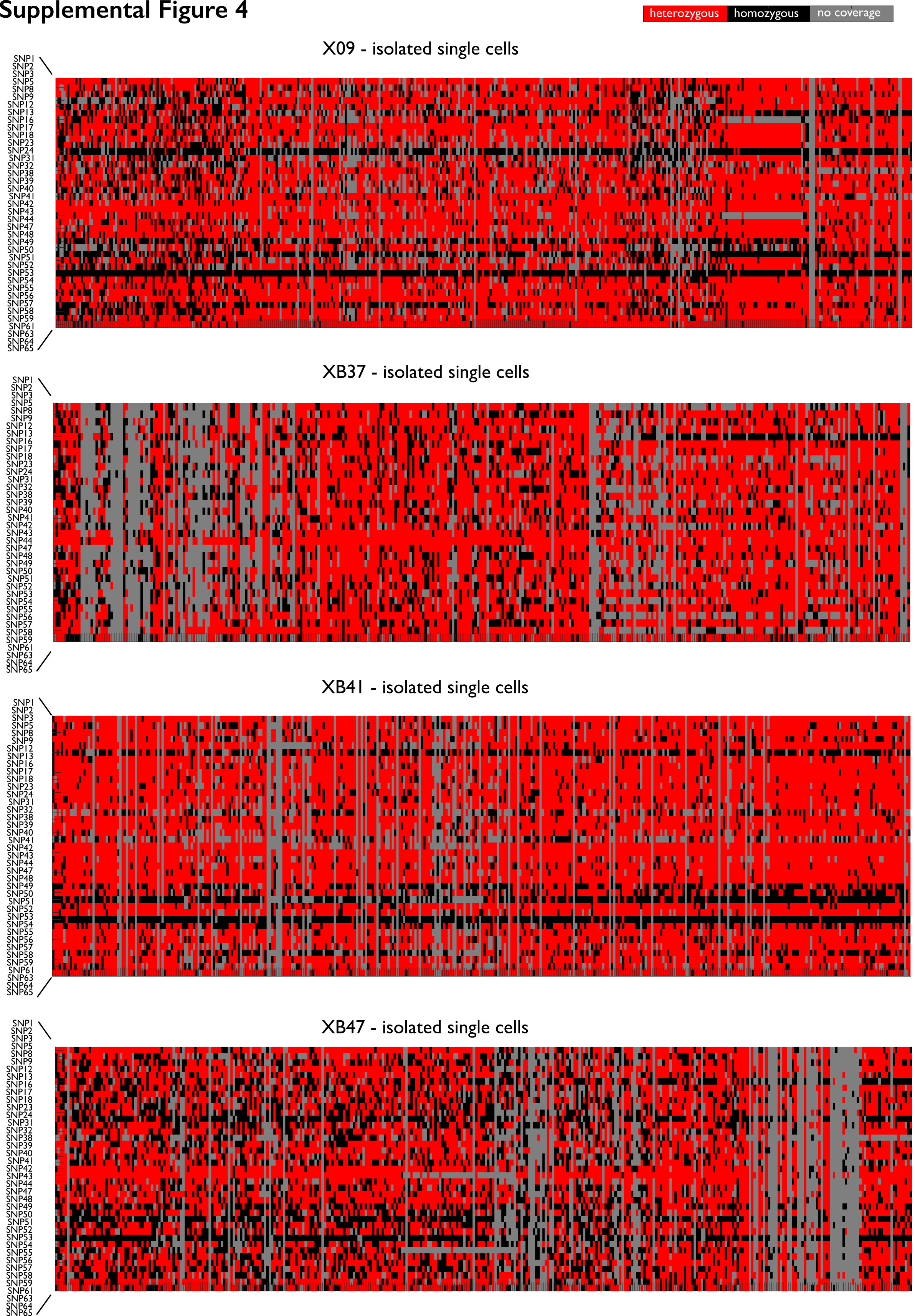

Supplement: Supplementary file 6 — Supplementary Figure 4 [file 41375_2018_127_MOESM6_ESM.jpg]

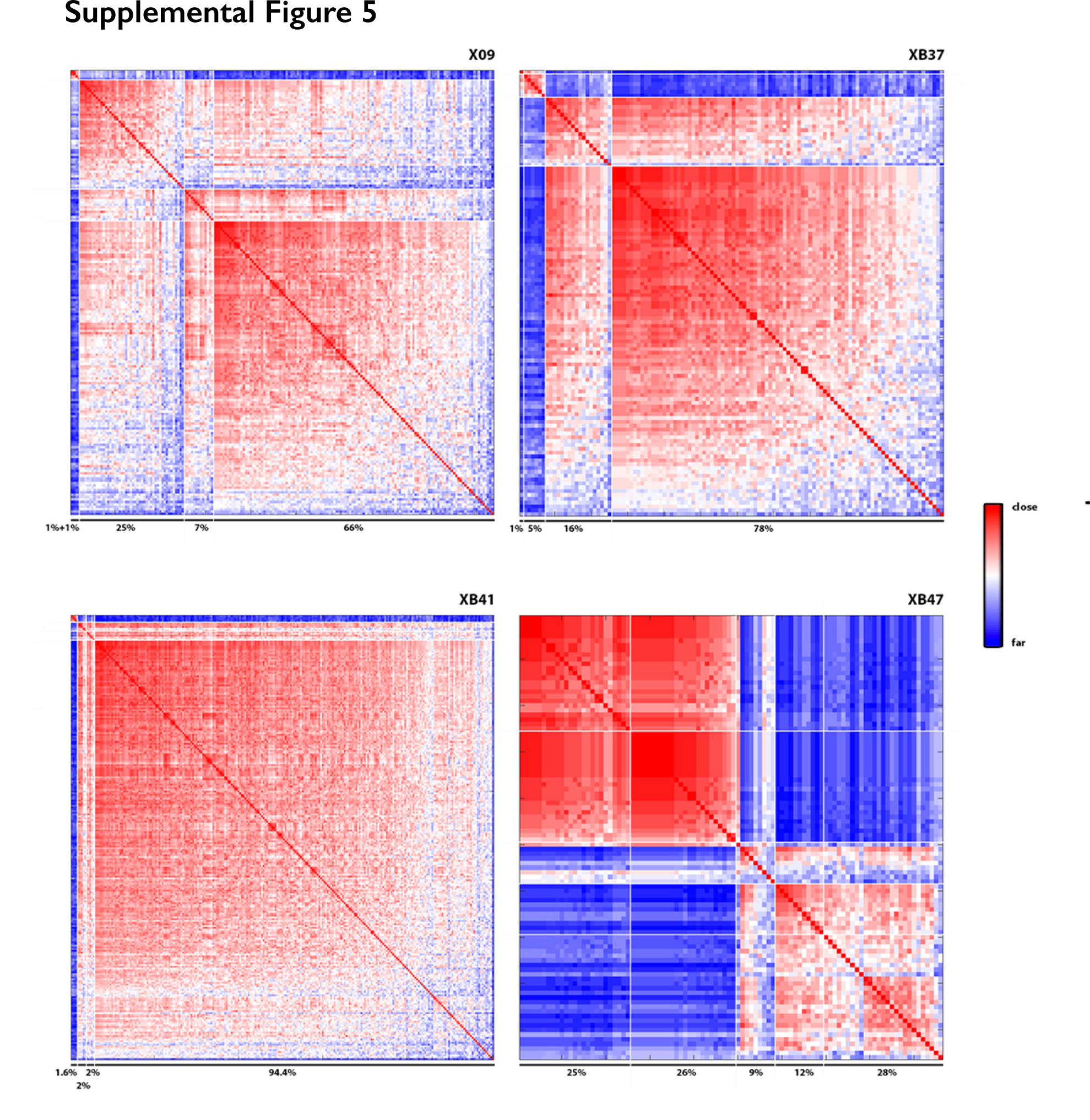

Supplement: Supplementary file 7 — Supplementary Figure 5 [file 41375_2018_127_MOESM7_ESM.jpg]

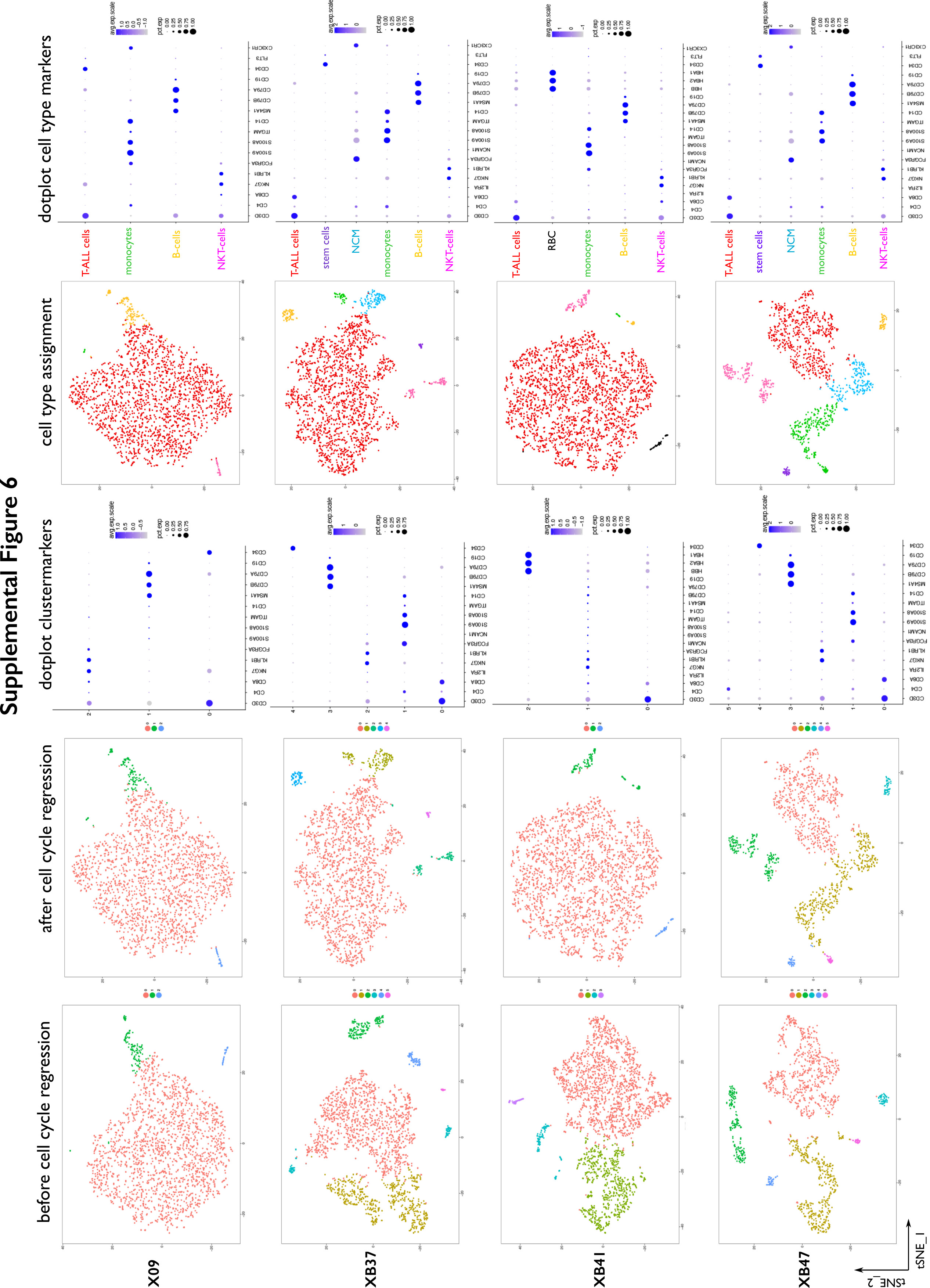

Supplement: Supplementary file 8 — Supplementary Figure 6 [file 41375_2018_127_MOESM8_ESM.jpg]

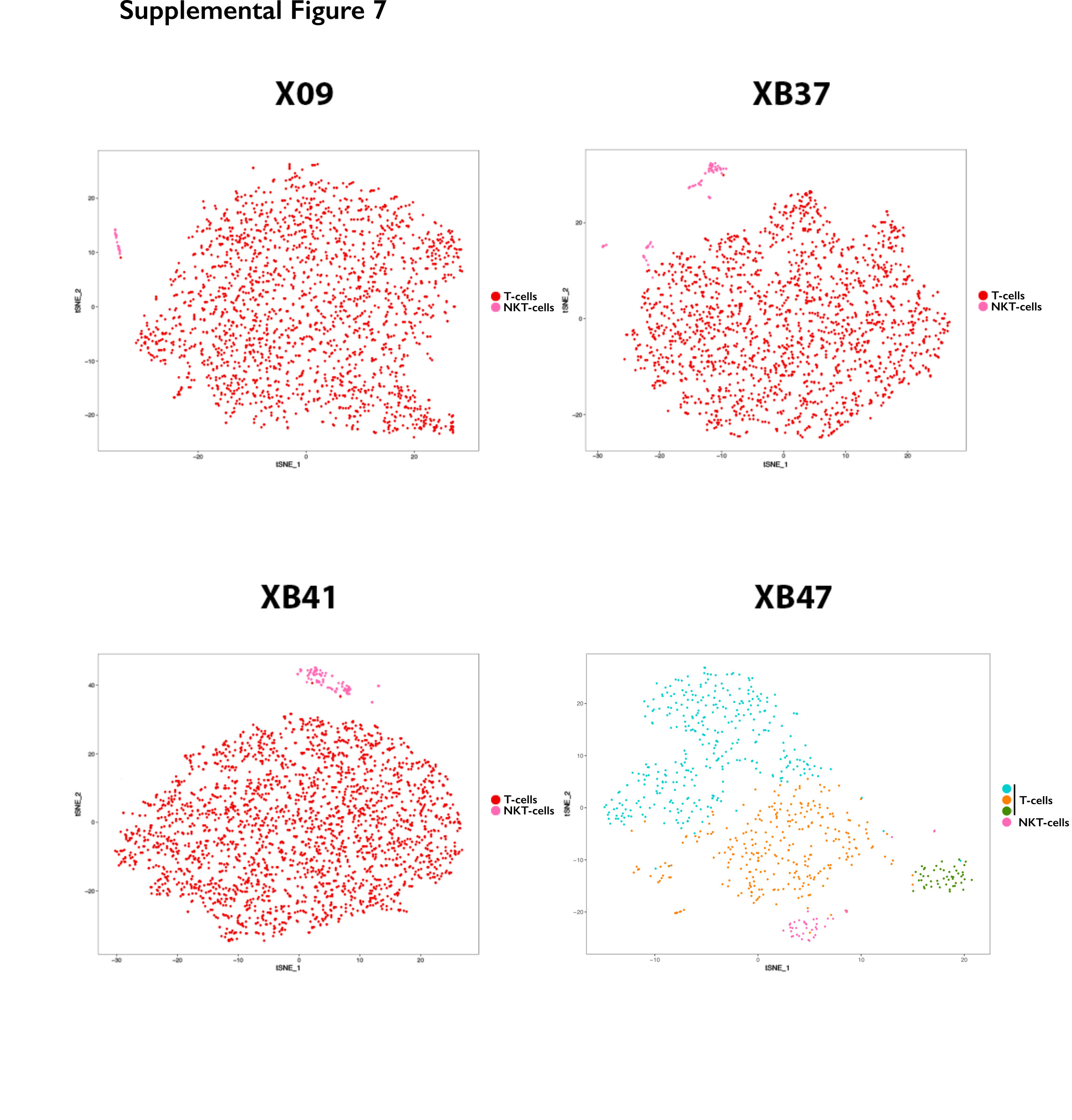

Supplement: Supplementary file 9 — Supplementary Figure 7 [file 41375_2018_127_MOESM9_ESM.jpg]

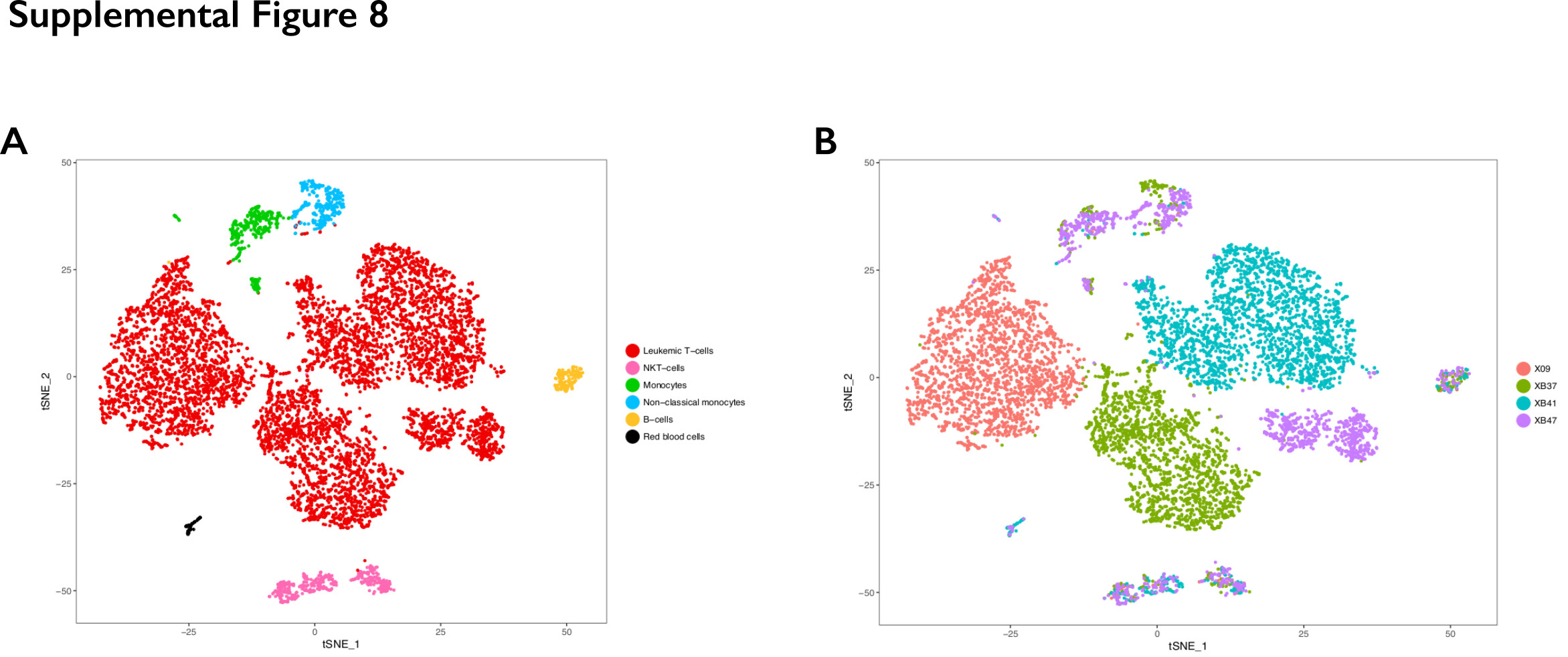

Supplement: Supplementary file 10 — Supplementary Figure 8 [file 41375_2018_127_MOESM10_ESM.jpg]

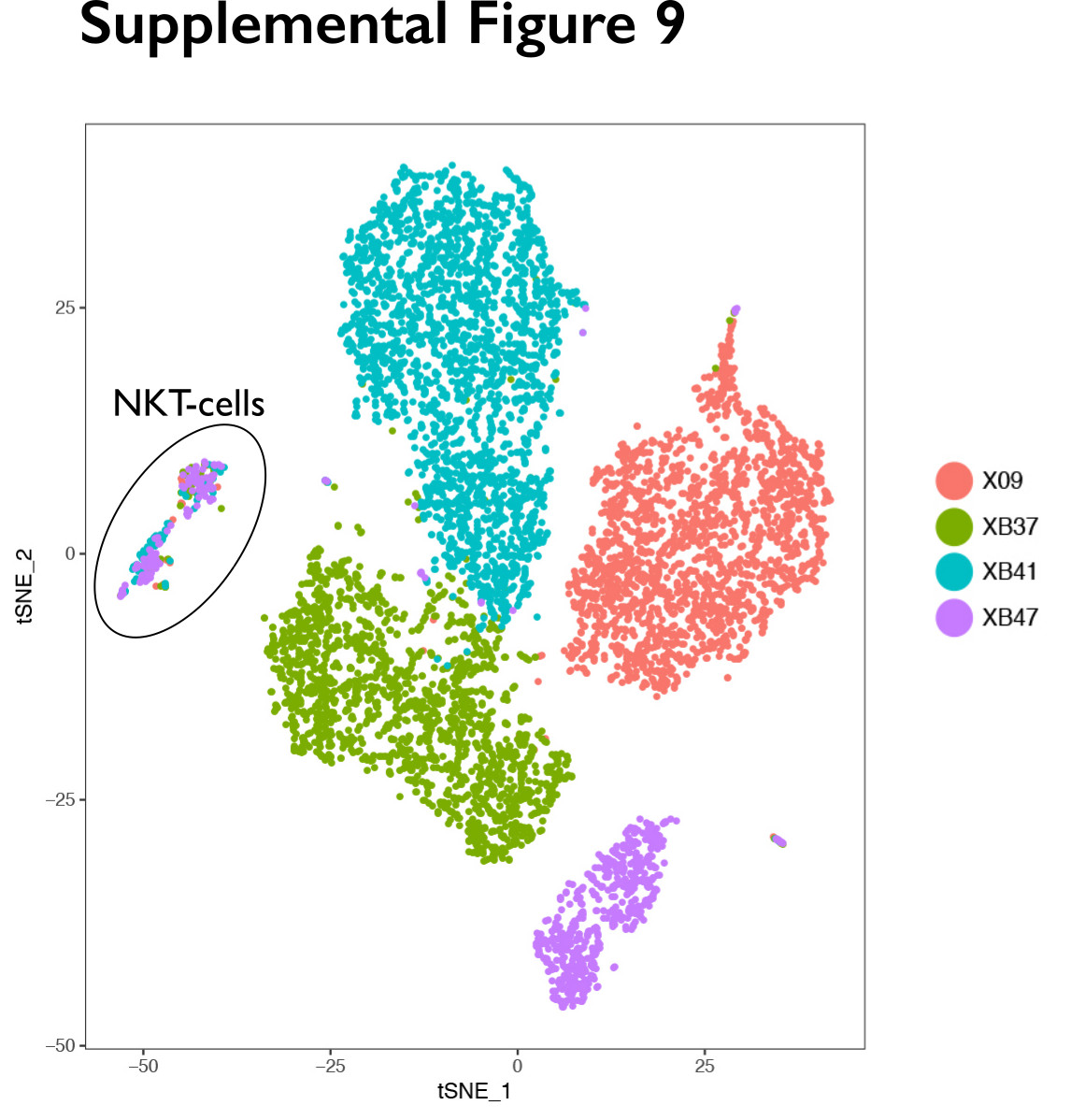

Supplement: Supplementary file 11 — Supplementary Figure 9 [file 41375_2018_127_MOESM11_ESM.jpg]

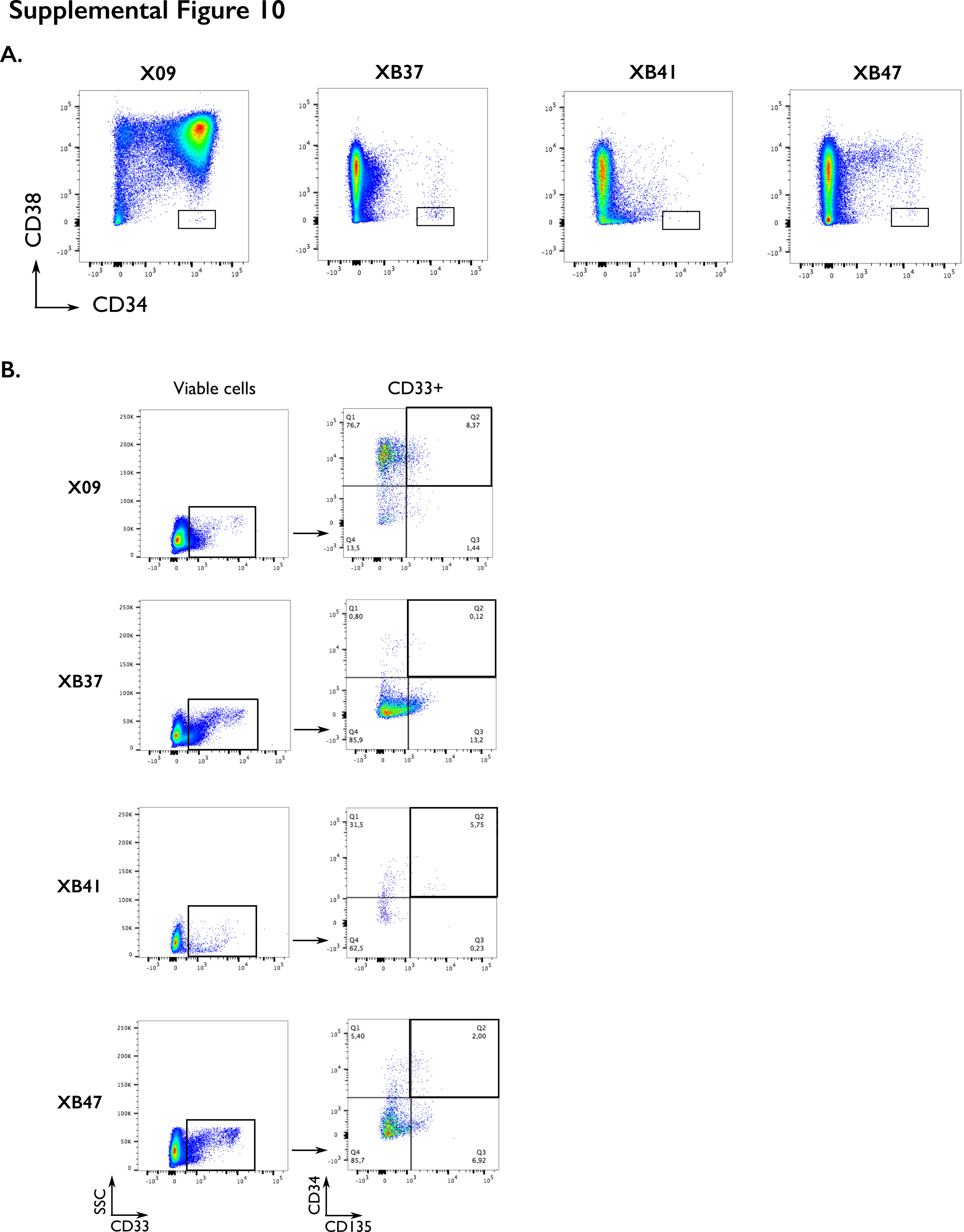

Supplement: Supplementary file 12 — Supplementary Figure 10 [file 41375_2018_127_MOESM12_ESM.jpg]
